# The Role of Early Nutritional Intervention in Preventing In-Hospital Complications Among Patients in the Medical Ward

**DOI:** 10.7759/cureus.89214

**Published:** 2025-08-01

**Authors:** Zia Ullah Khan, Muhammad Hamza, Khurrum Rashid, Hafiz Ali Shabbir Rajput, Areeba Inam, Talha Anwar, Safeer Mehmood, Ajab Khan, Muhammad Muttahir

**Affiliations:** 1 Medicine, Khalifa Gul Nawaz Hospital, Bannu, PAK; 2 Internal Medicine, Ayub Teaching Hospital, Abbottabad, PAK; 3 Medicine, Ayub Medical College, Abbottabad, PAK; 4 Surgery, National Health Service, England, GBR; 5 General Surgery, Ayub Medical College, Abbottabad, PAK; 6 Internal Medicine, Khyber Medical College, Peshawar, PAK; 7 Health and Sciences, University of Beira Interior, Covilhã, PRT; 8 Dentistry, Rehman College of Dentistry, Peshawar, PAK

**Keywords:** complications, early intervention, hospital stay, malnutrition, nutritional support

## Abstract

Introduction

Malnutrition is a common yet often overlooked issue in hospitalized patients, contributing to increased morbidity, prolonged hospital stays, and delayed recovery. Early nutritional intervention may help mitigate these risks.

Objective

This study aims to evaluate the role of early nutritional intervention in preventing in-hospital complications among medical ward patients.

Methodology

This retrospective observational study was conducted over a 12-month period at Khyber Teaching Hospital, Peshawar, Pakistan, and included 189 adult patients admitted to medical wards. Patients were divided into two groups: an intervention group (n = 95), which received both early nutritional screening (within 48 hours of admission) and at least one form of support (oral supplements, enteral feeding, or dietitian consultation), and a control group (n = 94), which did not receive such interventions. Data on demographics, nutritional status, interventions, and outcomes were collected. Statistical analysis included t-tests, chi-square tests, and logistic regression.

Results

Early nutritional support significantly reduced the incidence of hospital-acquired infections (12/95; 12.6% vs. 28/94; 29.8%, p = 0.004), pressure ulcers (4/95; 4.2% vs. 15/94; 16.0%, p = 0.009), and delayed wound healing (7/95; 7.4% vs. 19/94; 20.2%, p = 0.015). The intervention group also had a shorter mean hospital stay (7.2 ± 3.1 vs. 9.4 ± 4.6 days, p < 0.001). Logistic regression showed that absence of early nutrition and age > 60 years were independent predictors of complications.

Conclusion

Early nutritional intervention is linked to reduced complications and shorter hospital stays. Routine nutritional screening with timely support should be integrated into standard inpatient care.

## Introduction

Malnutrition remains a prevalent but often underrecognized issue among hospitalized patients, particularly those admitted to medical wards. It is estimated that up to 30%-50% of patients are either malnourished upon admission or at high risk of becoming malnourished during hospitalization [1]. This condition is associated with a wide range of adverse outcomes, including impaired wound healing, weakened immune response, prolonged length of stay, increased susceptibility to infections, and higher readmission and mortality rates [2]. Despite its significant clinical implications, nutritional status is frequently overlooked in the early phases of hospital care, leading to missed opportunities for timely intervention [3].

The causes of malnutrition in hospitalized patients are multifactorial. Chronic illnesses, acute medical conditions, anorexia, dysphagia, gastrointestinal disturbances, polypharmacy, and age-related physiological changes all contribute to a decreased nutritional intake and increased metabolic demands [4]. Institutional barriers such as delayed nutritional screening, limited dietetic support, and lack of awareness among healthcare providers exacerbate the problem [5]. Recognizing these contributing factors underscores the importance of implementing effective nutritional strategies at the onset of admission.

Early nutritional intervention, which includes timely screening, assessment, and initiation of individualized nutritional support, has gained increasing attention as a means to reduce complications and improve clinical outcomes [6]. Studies have shown that initiating nutritional support within the first 24-48 hours of hospitalization can reduce infection rates, enhance functional recovery, and shorten hospital stays [7]. Moreover, incorporating dietitians into multidisciplinary care teams can facilitate comprehensive management and monitoring of patients’ nutritional needs [8]. Yet, despite evidence supporting these benefits, early nutritional care is inconsistently applied in many healthcare settings, particularly in resource-limited environments [9].

In Pakistan and other developing countries, the implementation of structured nutritional care programs is further challenged by limited awareness, resource constraints, and inadequate integration of clinical nutrition into routine practice [10]. There is a critical need to explore the impact of early nutritional intervention in this context to generate locally relevant evidence and inform clinical practice. Despite global recognition of the importance of early nutritional care, limited data exist on its impact in Pakistani medical wards; therefore, this study aims to assess the role of early nutritional intervention in preventing in-hospital complications among medical ward patients.

## Materials and methods

Study design and setting

This retrospective observational study was conducted at Khyber Teaching Hospital, Peshawar, Pakistan, over a period of 12 months, from March 2023 to February 2024. The hospital serves a diverse patient population and has a dedicated nutrition department that maintains nutritional screening and intervention records. The study aimed to assess the impact of early nutritional intervention on the prevention of in-hospital complications among adult patients.

Sample size calculation

The sample size was calculated using the OpenEpi calculator for sample size, considering a confidence level of 95%, a margin of error of 5%, and an estimated prevalence of 50% for in-hospital complications among malnourished patients based on previous literature [11]. This yielded a minimum sample size of 174. To increase the power and account for possible incomplete records, a final sample size of 189 patients was selected for analysis.

Inclusion and exclusion criteria

All adult patients (aged 18 years and above) admitted to the medical wards during the study period were considered eligible for inclusion. Patients were excluded if they were admitted for less than 48 hours, were under palliative or end-of-life care, or had incomplete or missing nutritional or clinical records.

Group allocation was not randomized but was determined retrospectively based on hospital documentation of nutritional care. Patients were classified into the intervention group if they received both nutritional screening and at least one form of support (oral nutritional supplements, enteral feeding, or dietitian consultation) within the first 48 hours of admission. Early screening alone was not sufficient for inclusion in the intervention group. Those who did not receive nutritional intervention or received it after 48 hours were placed in the control group.

The decision to initiate nutritional support was based on clinical protocols, physician discretion, and dietitian availability. As this nonrandomized allocation could introduce bias related to illness severity or resource access, efforts were made to compare baseline characteristics such as age and comorbidities between groups. Additionally, binary logistic regression was used to adjust for potential confounding variables, including age, diabetes, and hypertension.

Data collection

Data were collected retrospectively from patient files, electronic medical records, and nutrition department documentation. Variables collected included demographic characteristics such as age and gender; relevant comorbidities, including diabetes and hypertension; the Charlson comorbidity index [12] as a measure of baseline illness burden; and the Barthel index [13] as a measure of functional status, where available. Nutritional risk was assessed using the Nutritional Risk Screening (NRS‑2002) [14] along with information about the type and timing of nutritional intervention. Clinical outcomes were also recorded, including discharge readiness, nutritional recovery status at discharge, and 30‑day readmission, where documented. Records with incomplete or missing critical data were excluded from the analysis to minimize bias and ensure data quality.

The primary outcomes assessed in the study were the incidence of in-hospital complications, which included hospital-acquired infections such as pneumonia and urinary tract infections, development of pressure ulcers, delayed wound healing, and unplanned readmissions during the same hospital stay. Secondary outcomes included the length of hospital stay and in-hospital mortality. Patients were divided into two groups for comparative analysis: the intervention group, which received nutritional assessment and support within 48 hours of admission, and the control group, which did not receive timely nutritional care.

Statistical analysis

Data analysis was performed using SPSS version 25.0 (IBM Corp., Armonk, NY). Continuous variables were summarized using means and standard deviations, while categorical variables were presented as frequencies and percentages. The normality of continuous data was assessed both visually and using the Shapiro-Wilk test. Group comparisons for continuous variables were performed using independent t-tests, and categorical variables were compared using chi-square tests. Binary logistic regression analysis was conducted to identify independent predictors of in-hospital complications. The regression model included early nutritional support, age, gender, diabetes mellitus, and hypertension as covariates to adjust for potential confounding. Results were expressed as adjusted odds ratios (AORs) with corresponding 95% confidence intervals (CIs). For outcomes where multiple comparisons were made (e.g., hospital-acquired infections, pressure ulcers, delayed wound healing, readmissions, and mortality), a Bonferroni correction was applied to control the family-wise error rate. The standard significance level of 0.05 was divided by the number of comparisons to determine adjusted thresholds for statistical significance in these analyses. Findings were interpreted based on the adjusted significance levels, where applicable. All statistical tests were two-tailed, and a p-value of <0.05 (after adjustment, where applicable) was considered statistically significant.

Ethical considerations

Ethical approval for the study was obtained from the Institutional Review Board (IRB) of Khyber Medical College, Peshawar, Pakistan (Approval No.: 928/DM/KMC). All data were de-identified to protect patient confidentiality and were used exclusively for this research.

## Results

The total sample included 189 patients, with 95 (50.3%) assigned to the intervention group and 94 (49.7%) to the control group. As summarized in Table 1, the mean age of the overall cohort was 57.6 ± 14.2 years, with a statistically significant difference between groups: patients in the intervention group were younger on average (55.8 ± 13.6 years) than those in the control group (59.4 ± 14.7 years, p = 0.047). Gender distribution was similar, with 103 males (54.5% of the total sample) and no significant difference between groups (50 males (52.6%) in the intervention group vs. 53 males (56.4%) in the control group). The prevalence of diabetes mellitus was 82 (43.4%) overall, equally distributed between groups (41 patients (43.2%) vs. 41 patients (43.6%), p = 0.954), while hypertension was present in 95 patients (50.3%), with no significant group difference (44 patients (46.3%) vs. 51 patients (54.3%), p = 0.270).

**Table 1:** Demographic and clinical characteristics of the study population (n = 189) *p-value < 0.05 was significant.

Variables	Total (n = 189)	Intervention group (n = 95)	Control group (n = 94)	Test used	p-value
Mean age, years	57.6 ± 14.2	55.8 ± 13.6	59.4 ± 14.7	t = 2.00	0.047*
Male, n (%)	103 (54.5%)	50 (52.6%)	53 (56.4%)	χ² = 0.26	0.611
Female, n (%)	86 (45.5%)	45 (47.4%)	41 (43.6%)	χ² = 0.26	0.611
Diabetes mellitus, n (%)	82 (43.4%)	41 (43.2%)	41 (43.6%)	χ² = 0.00	0.954
Hypertension, n (%)	95 (50.3%)	44 (46.3%)	51 (54.3%)	χ² = 1.22	0.270

**Figure 1:**
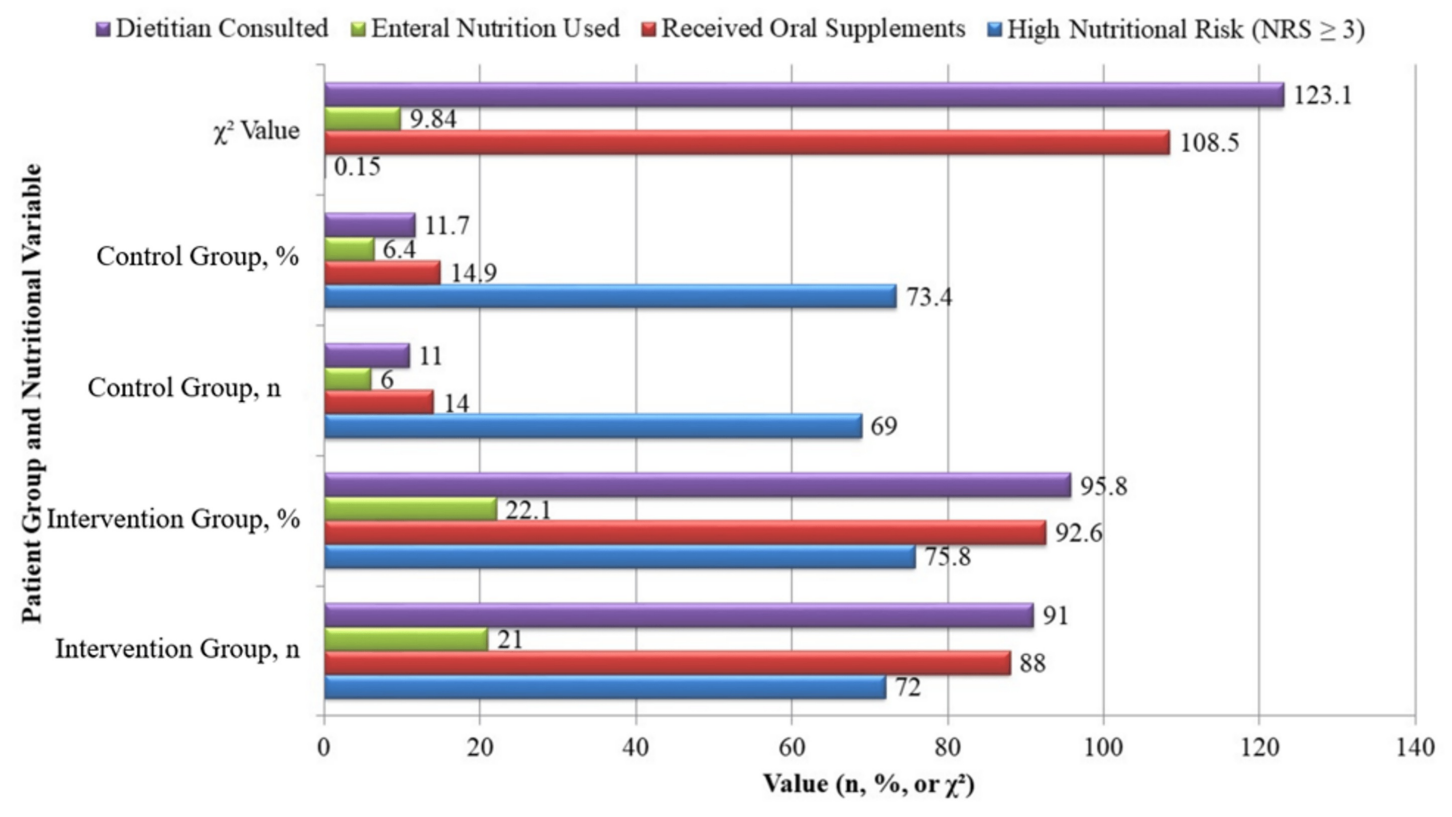
Nutritional risk screening and intervention details

Nutritional risk screening indicated that most patients were classified as high risk (NRS ≥ 3), with 72 patients (75.8%) in the intervention group and 69 patients (73.4%) in the control group (p = 0.704). However, the delivery of nutritional support differed markedly, as shown in Figure 1. Oral nutritional supplements were given to 88 patients (92.6%) in the intervention group and 14 patients (14.9%) in the control group (p < 0.001). Enteral feeding was initiated in 21 patients (22.1%) in the intervention group versus six patients (6.4%) in the control group (p = 0.002). Dietitian consultations were nearly universal in the intervention group (91 patients (95.8%)), while only 11 patients (11.7%) in the control group received this service (p < 0.001). These findings reflect high adherence to the nutritional intervention protocol in the intervention arm.

**Figure 2:**
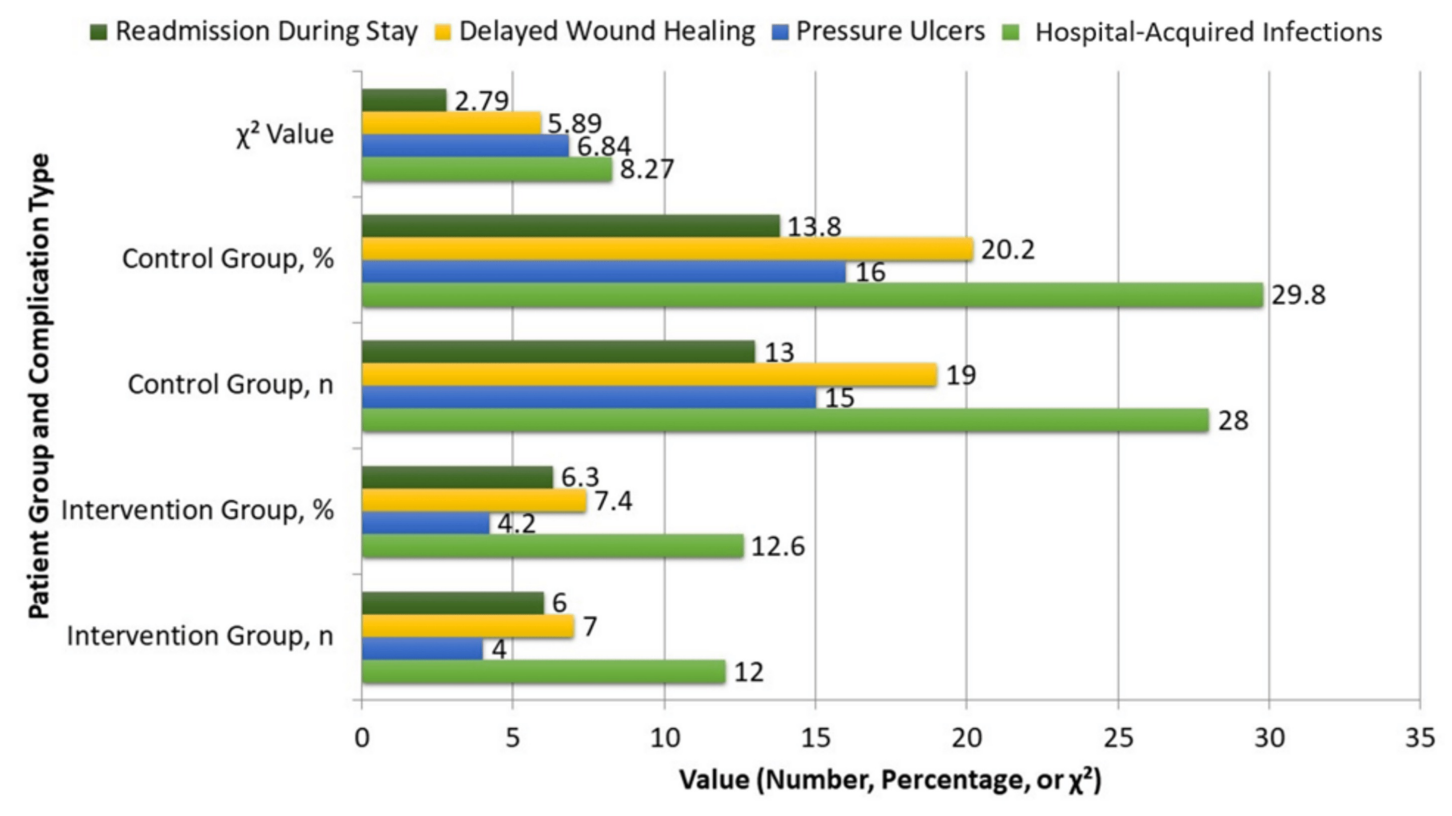
In-hospital complications between groups

As summarized in Figure 2, in-hospital complications were significantly less frequent among intervention patients. Hospital-acquired infections occurred in 12 patients (12.6%) in the intervention group compared to 28 patients (29.8%) in the control group (p = 0.004). Pressure ulcers developed in four patients (4.2%) in the intervention group versus 15 patients (16.0%) in the control group (p = 0.009). Delayed wound healing was noted in seven patients (7.4%) in the intervention group and 19 patients (20.2%) in the control group (p = 0.015). Although readmission was less frequent in the intervention group (six patients (6.3%) vs. 13 patients (13.8%)), the difference did not reach statistical significance (p = 0.095).

The average length of hospital stay was significantly reduced in patients who received early nutritional intervention, with a mean of 7.2 ± 3.1 days compared to 9.4 ± 4.6 days in the control group (t = 3.91, p < 0.001). This difference indicates a shorter and more efficient recovery among those receiving early care. In-hospital mortality occurred in three patients (3.2%) in the intervention group and nine patients (9.6%) in the control group (χ² = 3.23, p = 0.071). The chi-square test did not reach statistical significance (χ² = 3.23, p = 0.071), as shown in Table 2. This trend, however, suggests a potential survival benefit associated with early nutritional support.

**Table 2:** Length of stay and in-hospital mortality *p-value < 0.05 was significant.

Outcomes	Intervention group (n = 95)	Control group (n = 94)	Test used	p-value
Mean length of stay (days)	7.2 ± 3.1	9.4 ± 4.6	t = 3.91	<0.001*
In-hospital mortality, n (%)	3 (3.2%)	9 (9.6%)	χ² = 3.23	0.071

The multivariable logistic regression identified independent predictors of in-hospital complications after adjusting for age and comorbidities. As shown in Table 3, lack of early nutritional support was associated with higher odds of complications (adjusted OR: 2.68, 95% CI: 1.44-4.99, p = 0.002), and age over 60 years was also a significant predictor (adjusted OR: 1.91, p = 0.047). Diabetes mellitus and hypertension were not significant predictors. These findings reinforce the protective impact of timely nutritional support during hospitalization.

**Table 3:** Adjusted binary logistic regression model showing independent predictors of in-hospital complications Adjusted binary logistic regression model showing independent predictors of in-hospital complications. Model adjusted for age, diabetes mellitus, and hypertension. Other potential confounders, such as severity of illness, underlying diagnosis, and socioeconomic status, were not included due to data limitations. *p-value < 0.05 was statistically significant.

Variables	Adjusted OR	95% CI	p-value
No early nutritional support	2.68	1.44–4.99	0.002*
Age > 60 years	1.91	1.01–3.64	0.047*
Diabetes mellitus	1.63	0.89–2.99	0.113
Hypertension	1.28	0.70–2.36	0.418

## Discussion

This study demonstrated that early nutritional intervention in hospitalized medical patients significantly reduced the risk of in-hospital complications such as hospital-acquired infections, pressure ulcers, and delayed wound healing. It also led to a statistically significant reduction in hospital stay duration. While in-hospital mortality and readmission rates were lower in the intervention group, these differences were not statistically significant. Logistic regression analysis confirmed that lack of early nutritional support and age above 60 years were independent predictors of complications. These findings reinforce the clinical importance of integrating timely nutritional care in routine hospital management.

In this study, the intervention group was defined strictly as patients who received both early nutritional assessments within 48 hours and subsequent dietary support, ensuring that improvements in outcomes could be attributed to an active nutrition strategy rather than screening alone. The results of this study are in line with existing literature that highlights the role of early nutritional support in improving clinical outcomes among hospitalized patients. Similar studies have reported that malnutrition increases susceptibility to nosocomial infections, impairs wound healing, and prolongs hospital stay [15].

Early nutrition has been shown to enhance immune function, preserve lean body mass, and reduce inflammatory responses, all of which may contribute to the lower rates of complications observed in this study [16]. Moreover, our finding of reduced length of stay in the intervention group corroborates prior research demonstrating that prompt nutritional care shortens recovery time and improves cost-effectiveness in hospital settings [17]. The trend toward reduced mortality and readmission further supports the hypothesis that nutritional support plays a vital role in systemic resilience, though larger studies may be needed to confirm these associations [18].

Additional literature also suggests that timely identification of nutritional risk followed by targeted interventions - including oral supplementation, enteral nutrition, and professional dietary counseling - results in measurable improvements in clinical and functional outcomes [19]. Hospitals that adopt structured nutritional screening programs and standardized protocols for early intervention often report fewer complications and better patient satisfaction scores [20]. Furthermore, studies have emphasized that nutrition-related interventions are among the most underutilized yet cost-effective strategies in inpatient care [21].

Our findings resonate with these conclusions, particularly given the significant difference in the frequency of complications such as infections and pressure ulcers. This reinforces the critical importance of implementing institutional nutrition policies and routine screening mechanisms, especially in resource-limited healthcare settings where morbidity from preventable complications remains high [22].

Limitations and future suggestions

Despite the encouraging results, this study has several limitations. As a retrospective observational study, it is subject to information bias and cannot establish causality. The single-center setting in a tertiary care hospital may limit the generalizability of the findings to other institutions or populations with different characteristics. Nutritional status was assessed only at baseline using recorded screening tools, without reassessment during hospitalization, which may have affected the accuracy of risk classification. Although the regression analysis adjusted for age, diabetes mellitus, and hypertension, other potentially important confounding factors, such as baseline severity of illness, underlying diagnosis, and socioeconomic status, were not included in the model. This omission may have introduced residual confounding, which should be considered when interpreting the results. Furthermore, multiple outcome comparisons were conducted without correction for type I error (e.g., Bonferroni adjustment), which could have increased the likelihood of false-positive findings.

Future research should focus on multicenter, prospective cohort studies or randomized controlled trials to strengthen causal inference and enhance external validity. It is also important to examine the long-term effects of in-hospital nutritional support on post-discharge recovery, functional outcomes, and quality of life. Finally, integrating electronic nutrition monitoring systems and providing regular training for clinical staff could improve adherence to nutritional care protocols and further optimize patient outcomes.

## Conclusions

This study highlights the significant impact of early nutritional intervention on improving clinical outcomes in hospitalized medical patients. Timely nutritional support was associated with a reduction in complications such as infections, pressure ulcers, and delayed wound healing, along with a notable decrease in hospital stay duration. Although mortality and readmission differences were not statistically significant, the observed trends suggest potential long-term benefits. These findings underscore the importance of routine nutritional screening and early dietary management as integral components of inpatient care. Implementing structured nutrition protocols can enhance patient recovery and optimize healthcare resource utilization.
